# Supplementary material for: Are carbon emissions trading and green financial instruments synergistic? -Comprehensive quantitative research based on content analysis
Source: PLoS One. 2024 Mar 7;19(3):e0298601. doi: 10.1371/journal.pone.0298601 (PMC10919601; doi:10.1371/journal.pone.0298601)
Supplement: S1 Data — (ZIP) [file pone.0298601.s001.zip › Data/地级市Z_F工作报告.zip!/╣π╢1⁄2/╖≡╔╜╩╨/└┤╫╘╣1⁄2╓┌║┼í╛┬φ┐╦╩2╛▌═°í┐_2003.docx]

各位代表：

　　我代表市人民政府向大会作政府工作报告，请予审议，并请政协委员和其他列席人员提出意见。

　　过去五年工作回顾

　　本届政府任期五年来，坚持以邓小平理论为指导，努力实践“三个代表”重要思想，以率先基本实现社会主义现代化为总目标总任务统揽全局，以发展为主题，以结构调整为主线，以制度创新和科技创新为动力，在佛山市委的领导下，团结和带领全市人民与时俱进，开拓创新，全面完成“九五”计划和市十一届人大确定的各项任务，实现“十五”计划的良好开局，开创了我市改革开放和现代化建设的新局面。

　　综合经济实力跃上新台阶。五年来，国内生产总值以年均11.2%的速度增长，2002年达1168.66亿元（绝对值按当年价，增长率按可比价计，下同）；人均国内生产总值达34636元人民币，折4183美元。地方财政一般预算收入85亿元，比1997年增长1.68倍，五年年均增长21.8%。全市税收总额249亿元，比1997年增长1.7倍，五年年均增长21.9%。

经济结构调整取得新进展。所有制结构调整步伐加快。非公有经济尤其是民营经济发展迅速，民营经济占国内生产总值比重达41.8%，已成为推动我市经济发展的重要力量。产业结构进一步调整优化，一、二、三产业增加值比重由1997年的8.7∶54.7∶36.6调整为6.2∶53.1∶40.7。农业在结构调整中有效发展。农业总产值143.48亿元，五年年均增长6.2%。工业结构得到提升，优势行业迅速壮大。全社会工业总产值2703.88亿元，五年年均增长14.3%。第三产业发展层次进一步提高。第三产业增加值达475.44亿元，五年年均递增11.3%。市和南海被评为中国优秀旅游城市，旅游业成为重要的经济增长点。

　　各项改革实现新突破。公有企业转制面90%以上，其中涉及产权制度改革的占八成。加大国有企业扭亏脱困力度，基本实现扭亏脱困预期目标。推动了企业利用资本市场工作。构建了公有资产监管和运营体系。进一步完善社会保障体系，城镇职工养老和失业保险覆盖率以及基本医疗保险单位参保率都达95%以上，确保了国有企业下岗职工基本生活费和企业离退休人员养老金按时足额发放。行政管理体制改革得到深化和创新。推进政府管理企业方式改革，实施政府机构改革，行政审批制度改革取得阶段性成果。推行了预算管理体制改革和机关干部收入分配制度改革。同时，稳步推进粮食流通体制、农村股份合作制、农村合作医疗和农村税费改革。顺德作为全省综合改革试点，为全市改革提供了示范和经验。

　　国内外市场得到新拓展。五年来，在国内部分地区、香港地区举办和参加经贸洽谈及交流合作活动，提高佛山产品知名度和市场占有率。会展经济得到发展，成功举办陶瓷、家具、家电、花卉、内衣等重点产品专业博览会和库存商品交易会。积极调整消费结构，市场繁荣兴旺。2002年全社会消费品零售总额419.8亿元，五年年均增长12%。积极实施“外向带动”战略，优化调整出口结构，多元化出口市场格局初步形成。2002年进出口总额129.7亿美元，外贸出口五年年均增长12.3%，累计287亿美元。进一步优化投资环境，加大招商引资力度，利用外资质量和水平不断提高。五年累计实际吸收外资52.8亿美元，占历年总额的 45.1%。在扩大对外开放和交往中，外事、侨务和对台工作发挥了积极作用。

　　“科教兴市”取得新成果。坚持推动科技进步和技术创新，以企业为主体、产学研联合的技术创新体系初步形成。各级企业工程技术研究开发中心发展到97家，已有23家重点大学和科研院所与佛山企业进行科技合作。知识产权保护得到加强，累计专利授权量2.27万件、申请量2.94万件，均居全省前列。民营科技企业发展到500家，专业镇发展到15个，并且建立起一批生产力促进中心和工程技术服务中心。2002年，高技术产品产值605.9亿元，五年年均递增34.8%，占工业总产值比重达22.4%。高新技术企业发展到161家，其中国家火炬计划重点高新技术企业14家。我市被评为全国科技进步先进市。信息化建设取得显著成效。以信息技术普及和应用为重点，推进了信息产业化和产业信息化。2000年全面启动信息市建设，掀起信息化建设热潮，南海信息化建设得到党和国家领导人的充分肯定。电子政务建设稳步推进，信息化带动工业化步伐加快，省重点扶持的南海软件科技园呈现良好发展势头。我市被确定为全国制造业信息化工程重点城市和广东省信息化建设试点市，南海被确定为国家首批信息化试点城市和广东省信息化综合示范城市，顺德被确定为广东省制造业信息化试点市。2002年信息化综合指数达65%。教育改革和发展步伐加快。调整城乡中小学教育布局，改造一批薄弱学校，优化了教育资源配置。在巩固提高九年义务教育基础上，基本普及高中阶段教育，继续教育、职业教育和社会力量办学取得新进展。2002年高中毕业生升学率80.3%，适龄青年高等教育在校学生比例达到35.2%。全市已从小学三年级起逐步普及信息技术教育，加强了网络教育资源建设。初步建立起基础教育、职业教育、成人教育和高等教育协调发展的教育体系。顺德和南海被评为广东省教育强市。

　　基础设施和城镇化建设迈出新步伐。五年来，全社会固定资产投资总额达1039.44亿元，年均增长12.4%，完成一批重点基础设施建设项目。交通基础设施不断完善，全市通车里程达3974.6公里，公路密度104.22公里／百平方公里。各区在村村通公路的基础上基本实现村村通水泥路的目标。珠江三角洲城际快速轨道交通广州至佛山项目试验段工程建设进展顺利。电网建设满足社会经济发展要求，2002年各区实现城乡各类用电同网同价，农村住宅到户电价控制在0.75元／千瓦·时以内，全市总供电量达208亿千瓦·时。供水、燃气、污水处理设施建设取得新成绩。信息基础设施建设步伐加快。2002年底，全市移动电话用户305万门，本地电话用户150万门，固定电话及移动电话普及率分别为55部／百人和90部／百人，信息网络普及率为12%。水利建设进一步加强，五年建设了一批高标准、高质量、大规模的水利工程，并在堤防工程除险加固的基础上实施“路堤结合”。可持续发展战略全面实施，环境质量进一步改善。开展珠江流域及佛山水道和大气污染的综合整治工作，加快城镇生活污水处理进程，全市工业污染源基本实现达标排放。加快城市园林绿化建设，建成区绿化覆盖率34.05%，城镇人均公共绿地面积5.9平方米。我市先后被评为“国家园林城市”和“全国城市环境综合整治优秀城市”。城镇建设水平不断提高，中心城区功能逐步增强，具有珠三角地区特色的组团式城市群逐步形成，城市化水平达到59%。

　　依法行政和民主法制建设取得新成绩。五年来，完成“三五”普法教育，实施“四五”普法规划，全民法制观念进一步增强。推进基层民主政治建设，先后完成村委会换届选举和社区居委会民主选举工作，居民自治、村民自治逐步推行，城市社区建设继续发展。政府法制建设进程加快，行政执法水平得到提高。全面清理与世贸组织规则不相适应的规范性文件和政策措施，规范政府行为。进一步转变政府职能，加快了简政放权工作。在政府机关开展“高效优质服务”活动，政府部门的服务质量、服务水平和工作效率有新的提高。加强政府廉政建设，强化行政监察、审计和经济监督，推行政务公开和政府采购制度，政府职能和管理方式进一步转变。加强与工会、共青团和妇联等群众团体及工商联、各民主党派的联系，重视群众来信来访工作，民主渠道进一步拓宽。坚持向人大及其常委会报告工作、向政协通报情况，自觉接受监督。五年来共办理人大代表议案、建议和政协提案1231件。整顿市场经济秩序取得阶段性成果，制假售假、走私贩私、逃税骗税、逃汇骗汇等违法犯罪活动得到有效遏制。完善招投标制度，社会信用体系逐步建立，市场秩序、市场环境进一步得到规范和优化。强化安全生产管理，有效防止了重特大安全生产事故发生。加大地方金融资产清收力度，金融运行平稳。2002年金融部门存贷款余额分别比1998年初增长94.9%和57.8%。加强维护稳定及社会治安综合治理，开展了严打整治斗争，大力扫除“黄赌毒”等社会丑恶现象，坚决取缔“法轮功”邪教组织并做好痴迷者的转化工作，认真做好社会矛盾的调处工作。我市先后荣获“全国社会治安综合治理优秀单位”和“广东省维护稳定及社会治安综合治理先进市”称号。

　　社会各项事业呈现新气象。精神文明建设取得新硕果。深入开展学习邓小平理论、贯彻“三个代表”重要思想和“三讲”教育活动，全面贯彻《公民道德建设实施纲要》。以创建文明城市为龙头，开展多种形式的文明创建活动。市获得“广东省创建文明城市先进单位”称号，南海获得“全国创建文明城市先进单位”和“广东省文明城市”称号。文化建设加快发展，文艺创作喜获丰收，新闻出版、广播电视等文化事业为推进两个文明建设发挥了积极作用。群众性体育活动蓬勃开展，举办了市第五届运动会，承办了第九届全运会部分比赛项目，参加第十一届省运会，取得了好的成绩。稳妥推进医疗卫生体制改革，实施医疗机构分类管理，巩固和提高城乡初级卫生保健达标成果，广大居民健康水平得到提高。持之以恒抓好计划生育工作。坚持稳定低生育水平，提高人口素质，五年都圆满完成人口计划指标任务。国防建设和民兵预备役、拥军优抚工作得到进一步巩固和提高，市荣获全国双拥模范城“四连冠”，南海、顺德、三水、高明均成为广东省双拥模范单位。人民生活显著改善，总体上达到小康水平。2002年农民人均纯收入6288元，在岗职工年人均工资15872元，五年年均分别增长4.6%和8%；年末城乡居民储蓄余额1535.93亿元，五年年均增长12.5%。加快建立市场导向就业机制，切实做好下岗失业人员就业和再就业工作。2002年全市下岗失业人员安置率为60.3%，城镇登记失业率为2.56%。推进社会福利事业发展，健全和完善城乡居民最低生活保障制度，采取措施解决困难群众住房难、就医难、打官司难、子女入学难等问题。重视残疾人事业，殡葬改革工作稳步发展。宗教、人防、气象、保密、档案、物价、统计、老龄和对口扶贫等各项工作都取得新的成绩。

　　各位代表，过去五年是我市经济增长迅速、社会各项事业繁荣兴旺、人民生活殷实、现代化建设取得新成果的五年。回顾过去五年工作，我们的体会主要是：第一，必须把发展作为第一要务，积极探索和实践符合佛山实际的发展新思路，用发展的办法解决前进中的困难和问题。第二，必须坚持制度创新，深化各项改革，完善社会主义市场经济体制，大力促进非公有制经济尤其是民营经济的发展。第三，必须坚持科技创新，优化调整产业结构，坚定不移地走“产业强市”之路，用高新技术以及先进适用技术改造传统产业，大力发展高新技术产业，以信息化推动产业现代化，增强经济发展后劲。第四，必须以市场为导向，实施“外向带动”战略，加大招商引资力度，大力发展外向型经济，全面开拓国内外市场，拓展经济发展空间。第五，必须实施可持续发展战略，加强生态环境和城市建设，提高城市化水平，正确处理好经济建设与人口、资源、环境保护的关系，实现经济社会的协调发展。第六，必须坚持物质文明和精神文明建设同步推进，加强民主与法制建设，依法行政，大力发展先进文化，加强思想道德建设，促进社会各项事业全面进步。

各位代表，过去五年是不平凡的五年，我们经受了各种考验和挑战，如亚洲金融危机、世界经济增长放缓，以及原有改革和发展中积聚的深层次矛盾逐渐显现等等。为了解决前进中的困难和问题，本届政府认真履行职责，努力应对，克服困难，积极化解不利因素，经济和社会发展取得了来之不易的成效。这是在党中央、国务院和省委、省政府及市委正确领导下，是在市人民代表大会及常委会和市政协的监督支持下，是全市人民与时俱进、团结奋斗、共同努力的结果，是各民主党派、人民团体、驻禅部队及港澳台同胞、海外侨胞、外国友人和各界人士大力支持的结果。在此，我代表佛山市第十一届人民政府向所有参与、关心、支持佛山改革开放和现代化建设的同志们、朋友们，表示衷心的感谢和崇高的敬意！

在肯定成绩的同时，我们也清醒地认识到，我市经济和社会发展中还有不少困难和问题，主要是：经济结构还不尽合理，自主创新能力不强；结构调整和产业优化升级还有很多问题尚待解决；制约经济发展的体制性障碍和结构性矛盾仍然不同程度存在，发展环境尤其是软环境还不能令人满意；人民生活虽然总体达到小康水平，但发展不平衡，地区之间、城乡之间、不同阶层之间还有差距；市场经济秩序有待继续整顿和规范，社会治安环境还有待改善；行政区划调整后政府各部门的管理职能、管理方式、管理理念和管理行为有待进一步提高，社会管理新格局有待建立健全，困难群体的社会扶持力度仍需增大等等。对此，必须切实采取措施，认真加以解决。

今后五年奋斗目标及2003年工作建议

　　各位代表，回顾过去，豪情满怀；展望未来，任重道远。佛山的历史已翻开新的一页，佛山进入了新的发展阶段。今后五年是我市在新体制下全面建设宽裕小康社会、率先基本实现社会主义现代化的关键时期。今后五年政府工作的指导思想是：坚持以邓小平理论和“三个代表”重要思想为指导，全面贯彻党的十六大精神，按照省九次党代会以及省委九届二次全会和市委八届七次全会提出的战略部署，以胡锦涛总书记最近视察广东讲话的精神为动力，以发展为第一要务，以率先基本实现社会主义现代化为总目标总任务统揽全局，以提高城市综合竞争力为核心，以结构调整为主线，坚持走“产业强市”发展道路，继续深化改革和创新，进一步扩大对外开放，加快工业化、信息化和城市化步伐，全面推进建设广东省第三大城市、建设宽裕小康社会的进程，为建设经济强省、文化大省作出重要贡献。

　　今后五年奋斗目标：经济发展总量再上新台阶，国内生产总值年均递增9%左右，各项主要经济指标同步增长，经济发展质量和效益明显提高。团结和带领全市人民努力实现三大目标：一是在全省建设经济强省和文化大省中成为一支重要力量，作出重要贡献；二是在加快第三大城市建设中实现佛山传统历史底蕴与现代都市文明优势相融合，提升区域发展优势和国际竞争力。三是加快全面建设宽裕小康社会的进程，率先基本实现社会主义现代化。

　　今后五年主要任务：抓住机遇，充分利用现有条件，发挥优势，实现经济建设、城市建设和文化建设的更快发展。新型工业化要取得突破性进展，形成人口、资源、环境协调发展的新格局；积极探索创新城市化建设路子，推动城市化进程在现有城镇分布的基础上实现历史性新跨越；以教育、文化、科技、卫生、体育等事业为载体的现代城市文明建设呈现出生机勃勃的新态势，形成城市文明建设新亮点。

2003年是贯彻落实党的十六大精神的第一年，是我市行政区划调整后全面建设广东省第三大城市的第一年。做好今年政府工作，意义重大。

　　今年全市经济社会发展的主要预期目标是：国内生产总值增长10%，第三产业增加值增长10.5%，农业总产值增长5%，工业总产值增长12.5%，固定资产投资增长16%，出口总值增长8%，实际利用外商直接投资增长17%，居民消费价格水平持平或略有上升，社会消费品零售总额增长10.5%，城镇登记失业率控制在3.5%以内，人口自然增长率控制在6‰以下。

　　为实现上述任务和目标，对做好今年工作提出以下建议：

　　一、抓好各项规划的制定和实施，明确发展方向，优化资源配置

　　按照建设广东省第三大城市的要求，以及佛山在珠江三角洲经济区尤其是“广佛经济圈”所处的地位和作用，全力抓好各项规划的研究制定，明确今后发展方向。除要抓好已公布的交通发展规划、电网规划、产业发展规划以及教育发展规划的完善和实施外，还要在深入论证的基础上，科学制定城市发展概念规划、佛山市城镇体系规划和环境保护、文化建设以及其他基础设施建设等规划。加强指导，加大投入，积极组织各项规划的实施，促进资源有效配置。

　　二、确立产业强市战略，优化产业结构，提高经济发展整体素质

　　推进结构优化，走新型工业化的路子。积极实施产业强市战略，走出一条科技含量高、经济效益好、资源消耗低、环境污染少、人力资源优势得到充分发挥的新型工业化道路。组织实施《佛山市工业发展规划纲要》，大力推进产业结构的优化升级。把发展高新技术产业和以高新技术改造传统产业结合起来，重点发展电子信息、新材料、光机电一体化、生物医药和环保等高新技术产业，巩固提升家用电器、纺织服装、陶瓷及其他建材、金属材料加工及制品等支柱产业，加快发展装备制造业，壮大发展食品饮料、塑料制品、精细化工及医药、家居用品制造等潜力产业。大力实施名牌带动战略，培育一批具有自主知识产权和较强国际竞争力的名牌产品，构筑名牌群体优势。推进企业调整重组，支持一批科技型、品牌型的骨干企业发展壮大。加强对中小企业的扶持和服务，引导中小企业向“专、精、特、新”方向发展。

　　大力发展园区经济，加快产业承接和转移。抓好工业园区规划、建设和管理，加速工业企业向园区集中，促进工业发展集约化、簇群化。采取有力措施，在佛山国家高新技术产业开发区总体框架下，重点推进禅城高新技术产业开发区、顺德高新技术产业开发区、南海高新技术产业开发区、南海国家生态工业建设示范园区暨华南环保科技产业园、三水中心科技工业区、高明沧江工业园等主要工业科技园区的建设和发展，培育和形成各园区主导产业。按照产业发展导向，以园区为主要载体，承接国际和国内先进地区产业转移。将东南部的产业优势和西北部的地域优势充分结合起来，鼓励和支持西北部利用成本级差和发展空间优势，承接东南部产业转移，实现优势互补，加快经济发展。

　　以现代物流业为龙头，提升商品流通和服务行业的发展水平。突出发展面向本地优势产业的商品流通业和大力拓展社区居民生活服务业，提高第三产业在国民经济中的比重。要提升一批中高级专业批发市场，培育壮大一批连锁经营企业。积极发展为生产企业服务的第三方物流，完善物流信息平台。充分发挥佛山中国优秀旅游城市和历史文化名城优势，坚持开发历史文化资源和建设新兴旅游项目相结合，加快大旅游体系建设。围绕产业结构优化升级的需要，突出发展科研开发、金融、信息咨询服务等面向本地优势产业的服务业，积极培育教育、文化、卫生保健、体育产业和社区、公用服务业等第三产业新增长点。

进一步推进公有企业改革，完善公有资产管理体制。继续调整国有经济布局和结构，扩大公有资本退出领域。继续加大企业产权制度改革力度，积极稳妥地推进劣势企业退出市场。引导企业推行规范的公司制和股份制改革，完善法人治理结构。按照国家部署，推进国有资产管理体制改革，完善国有资产管理体系。规范资产经营公司和授权经营公司的监管营运，建立科学合理的公有资产经营业绩考核指标体系，提高公有资产管理水平。健全公有资产经营者激励与监管机制，规范推广年薪制，探索试行期权、期股制，鼓励劳动、资本、技术、管理等生产要素参与分配。

　　三、坚持外源型经济和内源型经济协调发展，增强发展动力

　　抓住我国加入世贸组织的有利时机，积极参与国际经济技术合作与竞争，大力发展开放型经济。拓宽外商投资领域，推动技术开发服务、零售、物流、旅游等第三产业对外开放。利用产业优势，积极吸引跨国公司和大财团直接投资，鼓励现有外商投资企业增资扩产，并在我市设立研发中心。迅速扩大利用外资规模，提高利用外资水平。积极引进高新技术项目和关联带动大的项目，促进产业结构优化调整和集约型经济的发展。创新招商引资方式，大力发挥行业协会、招商中介机构在招商引资中的积极作用。

　　积极实施出口多元化战略，增强产业国际竞争力。推进外贸经营主体多元化，协助民营、生产性企业申办进出口经营权。巩固提高加工贸易，大力发展自营出口。落实各项鼓励政策，支持重点出口企业扩大规模。推进出口产品多元化，促进名牌产品、机电产品、高新技术产品、高附加值产品出口，培育国际知名品牌。推进出口方式多元化，鼓励有条件的企业拓展境外加工贸易。吸引世界零售巨商到我市设立采购中心或连锁店，将佛山产品打入国际销售网络。加快“大通关”建设，运用电子信息技术，提高通关效率。

调动基层积极性，促进区、镇经济大发展。把经济发展重心下移，充分发挥各区在经济发展中的作用，采取强有力的措施，促使各镇成为经济发展主战场。进一步简政放权，要在市政府向区级政府简政放权的基础上，推动区级政府向镇级政府简政放权。强化中心镇在经济发展和加快城镇化建设的集聚功能，努力形成全市经济发展“虎虎生威”、“骏马腾飞”的新局面。

　　鼓励民间投资，做强、做大民营经济。按照“政治平等，政策公平，法律保障，放手发展”的方针，对民营企业“不限发展比例、不限发展速度、不限经营方式、不限经营规模”，进一步形成促进民营经济大发展的良好环境。放开市场准入条件，除国家法律法规和产业政策有规定外，各个行业和领域，都向民营企业开放。鼓励民间资本投资基础设施和社会公共事业。创造条件，重点支持科技型、外向型、吸纳下岗职工就业型和农产品加工型民营企业发展。切实解决民营经济发展中的各种难题，为民营企业提供融资、技术、信息、人才等方面的优质服务。引导民营企业加强技术创新、体制创新和管理创新，逐步建立现代企业制度，实现规模经营和集约经营。鼓励民营企业参与国有经济战略性调整，推动民营企业与国外资本有机结合。鼓励我市民营企业实施异地扩张，支持外地民营企业到我市投资创业。加强民营企业家队伍建设，努力使民营企业创业人员在“社会上有地位、政治上有荣誉、经济上有实惠”，开创民营经济大发展的新局面。

努力扩大消费需求，积极开拓国内市场。引导、支持企业拓展市场空间和企业发展空间。大力发展会展经济，依托产业优势，认真策划好家电、陶瓷、家具、花卉、纺织服装等专业博览会，推动企业努力拓展国内市场。加快商贸业发展，鼓励各区大力发展商贸业和饮食业，使我市成为区域性购物中心和美食文化聚集地。繁荣本地消费，改善消费环境，合理引导住宅、汽车等大宗消费，挖掘和培育新的消费热点。

　　四、加强农业和农村工作，千方百计增加农民收入

　　推进农业结构调整，大力发展效益农业，加快农业标准化、产业化进程。以都市农业为方向，发展优质、高产、高效、生态、安全农业。调整优化农业品种、品质结构，发展适时、适销、适应大中城市和出口需要的特色农产品。实施科技兴农战略，完善农业科技开发、推广和应用新机制。积极发展农业产业化经营，提高农民进入市场的组织化程度和农业综合效益。积极推行农业标准化生产，加快农产品质量安全体系建设，提高农产品在国内外市场的竞争力。进一步优化农业区域布局，建设一批有规模优势的农产品标准化生产基地和农业现代化园区。加大对农业龙头企业的扶持力度，通过龙头企业带动农户进行产业化经营。加强农业服务社会化体系建设，提高农业装备水平，大力发展农产品保鲜、包装、加工和储运服务。发展和完善农产品市场，进一步搞活农产品流通。

　　加快农村各项改革，改善农村发展条件。把发展农村二三产业与推进农村城镇化战略结合起来，加速农村劳动力向非农产业转移，增加农民的非农收入。大力推广农民公寓建设，抓好农村改貌工程的落实，改善农民居住条件和环境。按照依法、自愿、有偿的原则，推进土地承包经营权的合理流转，促进农村土地规模经营。在已实行农村股份合作制的地区，继续推广股权流转的有效方式。推进农村基层管理体制改革。加快农村管理信息化步伐，搞好村务财务公开。

　　多渠道增加农民收入，确保农村稳定。认真贯彻“多予、少取、放活”的方针，千方百计增加农民收入。加快建立农村社会保障体系，解决好农民最低生活保障和农村合作医疗等问题。加大对经济欠发达村和困难群众的帮扶力度，维护农村稳定。继续推进农村税费改革，抓好各项配套措施的落实，进一步清理农村各种不合理收费，切实减轻农民负担。加强对农村集体土地的管理，妥善处理好征地款的补偿和分配问题，切实保护农民的合法权益。

　　五、加快基础设施和生态环境建设，全面提高城镇化水平

　　实施城镇化战略，完善城镇发展体系。推进城镇化建设，提高城市化水平，优化结构、合理布局，以空间整合促进和带动城市产业、资源和基础设施的整合。明确佛山市发展战略定位，准确把握佛山市发展方向，按照现代化大城市发展思路、组团式城市发展布局，高标准编制城市总体规划、市域城镇体系规划和土地利用总体规划，明确市域内各城镇的地位、规模、发展方向和分工协作关系。做好全市重大基础设施规划以及各类工业园区的规划安排。落实扶持措施，全力支持中心镇建设，在户籍制度、土地使用制度、人事和机构体制、财税管理体制等方面制定有利于中心镇发展的政策，带动小城镇加快发展。发展小城镇要科学规划，合理布局，指导镇、村搞好规划，提高建设管理水平。

　　加大固定资产投资力度，高水平抓好基础设施建设。以交通道路和基础设施建设为龙头，以产业发展为重点，以工业园区和各镇为载体，多渠道增加固定资产投入，增强经济发展后劲。抓好主干公路交通网规划的实施，在加快珠江三角洲城际快速轨道交通广州至佛山段项目、新澜石大桥、三水二桥、高明大桥扩建等一批在建工程建设的同时，抓紧启动“一环”快速干线、“珠二环”高速公路、广三高速公路加宽、佛开高速公路加宽和佛明高速公路等以及广珠西线二期工程的建设。全面启动燃气管网建设项目。加强水利设施和农业基础设施建设，提高防灾减灾能力。继续推进城乡电网、信息网、轨道交通网等一批基础设施的规划和建设。同时，按照省运会的比赛要求，高标准规划和建设好一批大型体育场馆。认真整治城市道路交通，科学规划城市交通流向，抓好交通瓶颈路段的改造工作。

　　加强环境整治和生态保护工作。进一步落实环保目标责任制，切实改善城乡生态环境质量。以工业污染源全面达标为目标，推进工业污染源的深化治理，抓好陶瓷、纺织印染、水泥等重点污染行业的结构调整。组织实施好珠江综合整治工作，按计划推进佛山水道和西南涌、芦苞涌综合整治工程。加大大气污染综合治理力度，突出抓好二氧化硫、粉尘污染治理，逐步解决机动车尾气污染问题。在重要市政设施资源整合的基础上，加快建设一批污水处理厂、工业废水处理中心、垃圾处理场和危险废弃物处理中心等环保设施。抓好饮用水源保护，确保饮用水安全。大力推广清洁能源、清洁生产和废水回用、废物综合利用，发展循环经济。全面改善城市环境，搞好城市绿化、美化和亮化，展示良好的城市风貌。

　　切实提高城市经营管理水平。积极探索经营城市的有效方式，运用市场机制激活和运营城市资源，抓好城市公用事业改革步伐，实现城市公用事业投资多元化、运营企业化、管理社会化。在整合路桥收费站和邮政资源的基础上，整合城市公共资源，发展城市公交，实现区间交通公交化。利用电话号码升位的机遇，统一电话区号，降低电信区间收费。推进城市管理重心下移，充分发挥镇、街道和社区在城市管理中的积极作用。继续探索城市管理综合执法，形成各层次职能分工明确的城市管理综合执法机制。加大对“城中村”的整治、改造力度，坚决查处违章违法建设行为。

　　六、实施“科教兴市”战略，加快推进信息化建设步伐

　　加强技术创新体系建设，增强企业技术创新能力。坚持开展经常性、群众性、社会性的科学普及活动，不断提高全民的科学文化素质。继续引导企业建立健全工程技术研发机构，支持骨干企业在关键领域中掌握具有自主知识产权的核心技术。加强各级生产力促进中心、专业镇创新中心和行业技术服务中心建设，发展技术市场，建立面向广大中小企业的社会化创新服务体系。深入开展产学研合作，探索多样化合作模式，提升合作层次。加强对高新技术产业项目的技术支持，多方式推动企业利用高新技术和先进适用技术改造传统产业。探索建立科技风险投资机制，促进科技成果转化，支持科技型中小企业的发展。积极实施专利战略，加强知识产权保护工作。

　　深化教育改革，构建终身教育体系。以建设教育强市为目标，构建现代终身教育体系，形成全民学习、终身学习的学习型社会，促进人的全面发展。优化配置教育资源，继续调整中小学布局，提高教育质量与效益。全面实施素质教育，加快推进中小学信息技术教学和英语教学，巩固提高普及九年义务教育和基本普及高中阶段教育成果。大力发展面向全社会的职业教育，因地制宜发展成人教育、社区教育和老年教育。加快大学园区建设，吸引更多国内外著名高校和研究院（所）到佛山办学，提升高等教育发展层次。推行办学体制改革，鼓励社会力量办学，形成以政府为主导，公办与民办学校共同发展的格局。推进教育信息化，加快教育技术和教育手段现代化，抓好一批示范性学校建设。

　　加大人才引进和培育力度，鼓励人才创业。积极实施人才战略，抓好人才资源的储备和开发。设立人才培育专项资金，探索人才培育新途径。取消人才准入限制，落实好人才引进的各项优惠政策。优先引进国内外高层次人才，特别是留学归国人员和各类科技、学术带头人。完善人才激励机制，探索人才评估和薪酬分配制度改革的有效方式。建立人才创业服务体系，提供人才创业的良好平台。

　　加快推进信息化建设，提升国民经济和社会信息化水平。以电子政务建设为重点，推动信息技术在社会各领域的广泛应用。在“规划、应用、整合、管理”上下功夫，确保信息化建设有序、健康发展，促进资源共享。加强电子政务规范化管理，全面提高政务信息化应用水平。推进制造业信息化示范工程，引导和协助企业依靠信息技术提高研发、设计、制造和管理水平。建立健全与电子商务配套的认证体系和网上支付体系，促进电子商务发展。以南海软件科技园和骨干企业为依托，以软件及系统集成业、数字多媒体广播产业为重点，加快信息产业发展。

　　七、构建公共财政体系，强化金融监管

　　规范财政收支管理，构建公共财政体系。严格依法治税，强化税收征管，严厉打击各种偷逃骗税行为。积极培养财源，确保财政稳步增收。优化调整财政支出结构，确保政府机关正常运转，以及社会保障、科技教育、环境保护、基础设施、农业水利、文化建设等公共事业的支出。继续推行委派财务总监制度，加强对各项财政性专项资金的监管，建立财政资金追踪问效管理机制。改革预算编制方式，全面推行部门预算，严格执行“收支两条线”规定，建立、完善综合财政预算。完善财政预算执行机制，推进财政国库管理制度改革。依法健全和规范政府采购制度，强化对政府采购行为的监管，扩大政府采购范围。改革投融资体制，改进财政资金对产业发展的扶持方式。

　　维护金融运行秩序。加强对地方金融机构的监管，严厉打击逃废银行债务等金融违法犯罪行为。加强金融机构信息沟通，健全金融风险预警机制。引导金融机构调整信贷结构，加大对非公有经济、中小企业和基础设施建设的扶持力度。

　　八、推进信用体系建设，优化投资发展环境

　　整顿和规范市场秩序。抓好政府信用建设和企业信用建设工作。按照“统一、开放、竞争、有序”的原则，健全现代市场体系。逐步取消不必要的市场准入限制，鼓励规范有序的市场竞争行为。进一步完善土地、建筑工程和劳动力市场，规范发展产权、技术等要素市场。坚持治理整顿与制度建设并举，严厉打击破坏市场经济秩序的违法犯罪活动。整顿和规范文化市场秩序，加强对互联网上网服务和娱乐场所的管理。坚决落实安全生产责任制，推行注册安全主任和班组安全员等项制度，建立长效的安全生产管理机制。

进一步优化投资环境。以制度完善和设施配套为重点，落实优化投资环境各项措施。进一步规范收费行为，实施收费公示制度，取消不合理收费。在公用事业领域推行价格听证制度，建立成本约束机制。根据产业布局和结构调整需要，降低工业地价。推行“一站式”服务和办事承诺制、首问责任制、一个窗口受理制等方便快捷的办事方式，努力办好各级行政服务中心，提高办事效率和服务质量。培育发展各类中介机构，建立专业化分工、社会化服务的中介服务体系。

　　加强社会治安综合治理。落实社会治安综合治理目标管理责任制，加强治安防控体系建设，建立健全社会治安管理长效机制。深化“三警合一”警务机制改革，大力开展社区警务建设，推进社区群防群治工作。继续深入开展严打整治斗争，严厉打击各种暴力犯罪、带黑社会性质的有组织犯罪和多发性犯罪。严密防范“法轮功”邪教组织的破坏活动，坚决扫除“黄赌毒”等社会丑恶现象。做好基层调解工作，妥善处置各类群体性事件。

　　九、发展先进文化和社会各项事业，提高人民群众生活质量

　　深入开展精神文明创建活动。坚持以“三个代表”重要思想统领文化建设，组织广大干部群众深入学习党的十六大精神。坚持把依法治市和以德治市结合起来，认真贯彻《公民道德实施纲要》。继续抓好普法工作，提高市民文明素质。深入开展多形式的精神文明创建活动，塑造文明城市形象。

推进文化体制改革，发展文化事业和文化产业。发挥国家历史文化名城的优势，坚持继承和创新相结合，弘扬优秀传统文化，发展现代都市文化，增强城市文化综合竞争力。加强文化服务体系建设，高起点、高标准建设一批文化设施。强化文物保护工作，提高历史文化街区开发利用水平。实施文化精品战略，繁荣文艺创作。推进文化体制改革，促进文化艺术机构在改革中发展壮大。积极发展文化产业，多渠道满足市民文化需求。

　　发展体育卫生事业，抓好第十二届省运会筹备工作。抓好竞赛和各项比赛项目的竞技训练，推动全民健身运动广泛开展。推进体育事业改革，发展体育产业。优化配置医疗卫生资源，完善多层次的医疗卫生服务体系。加强农村医疗机构和卫生队伍建设，加快推进镇村卫生机构一体化管理。大力开展社区卫生服务，健全社区卫生服务网络。加强卫生监督工作，建立和完善疾病预防控制系统，有效控制、预防急性传染病和食物、职业中毒事件。推行和完善药品集中招标采购制度，减轻群众医药费用负担。完善医疗机构分类管理制度，规范发展营利性医疗机构，培育和发展卫生保健产业。当前，要在党中央、国务院和省委、省政府的坚强领导下，团结一致，众志成城，夺取我市抗击非典型肺炎防治工作的全面胜利。

　　加强计划生育和双拥工作，推进城市社区建设。落实人口与计划生育目标管理责任制，控制人口增长，稳定低生育水平，强化人口发展战略。加强计划生育基层基础工作，积极推进计划生育村（居）民自治。改革计划生育工作机制，强化城市计划生育属地管理和流动人口计划生育现居住地管理。积极开展以技术服务为重点的计划生育优质服务，建立健全计划生育利益导向机制，逐步转变全社会的生育观念。重视民兵、预备役工作，完善防空设施的建设和管理，抓好全民国防教育。坚持做好拥军优属工作，争创全国双拥模范城“五连冠”。继续推进社区建设，建立新型的社区自治工作机制。深化城市基层管理体制改革，拓展社区服务领域，完善社区服务体系，促进社区资源共享。

　　千方百计扩大就业。加强宏观调控，调整就业结构，拓宽就业渠道，推行灵活多样的就业形式，广开就业门路。健全就业援助机制，认真抓好再就业困难群体的再就业工作。加强职业技能培训和创业培训，发展公益性职业介绍机构和劳动力市场，完善公共就业服务体系。加大劳动监察力度，维护劳动者合法权益。

建立与经济发展水平相适应的社会保障体系。以基金收支平衡为核心，提高社会保障制度运行水平，巩固扩面成果，强化基金征缴，完善城镇职工基本养老、失业、工伤和基本医疗保险制度。多渠道筹集资金，确保国有企业下岗职工基本生活费和企业离退休人员基本养老金按时足额发放。完善社会保障各项资金的统筹管理，增强保障能力。全面构建社会保障社区工作平台，企业退休人员逐步实现社会化管理。

进一步巩固以低保制度为核心的社会救助体系。对困难群体加强扶持，搞好城乡社会救济和社会福利事业。建立健全城乡居民最低生活保障制度，实现应保尽保。加快完善医疗救助、教育救助、廉租房和法律援助等制度，切实解决困难群众“四难”问题。重视残疾人工作，积极发展老龄事业。

　　十、推进政治文明，加强政府自身建设

　　规范行政行为，促进依法行政。坚持向人大及其常委会报告工作、向政协通报情况的制度。落实《佛山市人大常委会讨论决定重大事项规定》，切实办理好人大议案、人民代表建议和政协提案。认真听取各民主党派、工商联和无党派人士的建议，充分发挥工会、共青团、妇联等人民团体的桥梁和纽带作用。规范各级政府行政行为，多形式加强对行政行为的监督，促进依法行政。加大行政执法监督力度，完善和落实行政执法责任制、重大行政处罚备案审查制度。加强行政执法队伍建设，增强行政公职人员依法办事能力。认真做好行政复议和行政应诉工作，高度重视人民来信、来访，妥善解决群众关心的热点难点问题。

　　深化行政管理体制改革，加快政府职能转变。以建立行为规范、运转协调、公正透明、廉洁高效的行政管理体制为目标，推进行政管理体制改革。结合行政区划调整，进一步实施简政放权，促进政府职能加快转变。将市一级政府的主要职能转移到规划、调研、策划、督办和监管上来，形成各级政府职能分工明确、层次少、效率高、服务好的行政管理体系。深化行政审批制度改革，减少审批事项，规范审批行为。在机关开展以“三创新一优化”创新观念、创新体制、创新服务和优化发展环境为主题的机关作风建设年活动，强化服务意识，提高行政效能和服务质量。

　　积极推进政务公开，完善民主决策机制。健全政务公开机制，促进政务公开信息化、规范化、制度化，建设“阳光政府”。通过电子政务网络，建立方便快捷的政策发布、信息查询、社会监督渠道。完善政府决策的程序和制度，建立健全决策咨询、重大决策及建设项目社会参与讨论、社会听证等制度，加快形成民主决策、科学决策、依法决策机制。

　　深化干部人事制度改革，提高公务员队伍素质。继续推进干部人事制度改革，完善公务员竞争上岗、交流轮岗和考核制度。落实选优汰劣、能进能出的机制，优化公务员队伍结构。抓好公务员培训，鼓励公务员接受继续教育，提高公务员队伍整体素质。深化事业单位体制改革，全面推进全员聘用制。加大行政效能监察和审计监督力度，搞好政府系统廉政建设，抓好机关纠风工作，落实廉洁自律规定，深化改革、加强教育、创新体制，从源头上预防和治理腐败。

　　各位代表！前进的方向已经明确，历史和时代已赋予我们重任。我们要以邓小平理论和“三个代表”重要思想为指导，全面贯彻党的十六大和省委九届二次全会精神，聚精会神搞建设，一心一意谋发展，为全面建设宽裕的小康社会，加快率先基本实现社会主义现代化而努力奋斗！

立行为规范、运转协调、公正透明、廉洁高效的行政管理体制为目标，推进行政管理体制改革。结合行政区划调整，进一步实施简政放权，促进政府职能加快转变。将市一级政府的主要职能转移到规划、调研、策划、督办和监管上来，形成各级政府职能分工明确、层次少、效率高、服务好的行政管理体系。深化行政审批制度改革，减少审批事项，规范审批行为。在机关开展以“三创新一优化”创新观念、创新体制、创新服务和优化发展环境为主题的机关作风建设年活动，强化服务意识，提高行政效能和服务质量。

　　积极推进政务公开，完善民主决策机制。健全政务公开机制，促进政务公开信息化、规范化、制度化，建设“阳光政府”。通过电子政务网络，建立方便快捷的政策发布、信息查询、社会监督渠道。完善政府决策的程序和制度，建立健全决策咨询、重大决策及建设项目社会参与讨论、社会听证等制度，加快形成民主决策、科学决策、依法决策机制。

　　深化干部人事制度改革，提高公务员队伍素质。继续推进干部人事制度改革，完善公务员竞争上岗、交流轮岗和考核制度。落实选优汰劣、能进能出的机制，优化公务员队伍结构。抓好公务员培训，鼓励公务员接受继续教育，提高公务员队伍整体素质。深化事业单位体制改革，全面推进全员聘用制。加大行政效能监察和审计监督力度，搞好政府系统廉政建设，抓好机关纠风工作，落实廉洁自律规定，深化改革、加强教育、创新体制，从源头上预防和治理腐败。

　　各位代表！前进的方向已经明确，历史和时代已赋予我们重任。我们要以邓小平理论和“三个代表”重要思想为指导，全面贯彻党的十六大和省委九届二次全会精神，聚精会神搞建设，一心一意谋发展，为全面建设宽裕的小康社会，加快率先基本实现社会主义现代化而努力奋斗！
